# Mapping the global research landscape on malnutrition for patients with chronic kidney disease: a visualization analysis

**DOI:** 10.1186/s41043-023-00445-8

**Published:** 2023-09-23

**Authors:** Muna H. Shakhshir, Divya Vanoh, Mohanad Hassan, Sa’ed H. Zyoud

**Affiliations:** 1 Department of Nutrition, An-Najah National University Hospital, Nablus, 44839 Palestine; 2 Department of Public Health, College of Medicine and Health Sciences, An-Najah National University, Nablus, 44839 Palestine; 3 Dietetics Programme, School of Health Sciences, Universiti Sains Malaysia, Kelantan, Health Campus, 16150 Kubang Kerian, Malaysia; 4 Department of Medicine, College of Medicine and Health Sciences, An-Najah National University, Nablus, 44839 Palestine; 5 Department of Nephrology, An-Najah National University Hospital, Nablus, 44839 Palestine; 6 Poison Control and Drug Information Center (PCDIC), College of Medicine and Health Sciences, An-Najah National University, Nablus, 44839 Palestine; 7 Department of Clinical and Community Pharmacy, College of Medicine and Health Sciences, An-Najah National University, Nablus, 44839 Palestine; 8 Clinical Research Centre, An-Najah National University Hospital, Nablus, 44839 Palestine

**Keywords:** Chronic kidney disease, Bibliometric, Malnutrition, Scopus, VOSviewer

## Abstract

**Background:**

Chronic kidney disease (CKD) is seen as a diverse disease and a primary contributor to global mortality. Malnutrition arises within chronic illness, which involves protein energy depletion and inadequate levels of essential nutrients. These factors increase the likelihood of death and the overall impact of the disease on affected individuals. Consequently, this study aims to utilize bibliometric and visual analysis to assess the current state of research, the latest advances and emerging patterns in the fields of CKD and malnutrition.

**Methods:**

Extensive research was conducted using the Scopus database, which is the most authoritative database of research publications and citations, to focus on CKD research between 2003 and 2022, as indicated by title and author keywords. Then, within this vast collection of academic publications, a notable subset of articles was exclusively dedicated to investigating the relationship between CKD and malnutrition. Finally, we performed bibliometric analysis and visualization using VOSviewer 1.6.19 and Microsoft Excel 2013.

**Results:**

Large global research between 2003 and 2022 resulted in 50,588 documents focused on CKD, as indicated by title and author keywords. In this extensive collection of scientific publications, a staggering portion of 823 articles is devoted exclusively to investigating the link between CKD and malnutrition. Further analysis reveals that this body of work consists of 565 articles (68.65%), 221 reviews (26.85%), and 37 miscellaneous entries (4.50%), which encompass letters and editorials. The USA was found to be the most productive country (*n* = 173; 21.02%), followed by Italy (*n* = 83; 10.09%), Sweden (*n* = 56; 6.80%), Brazil (*n* = 54; 6.56%) and China (*n* = 51; 6.20%). The most common terms on the map include those related to the topic of (a) malnutrition in hemodialysis patients and predicting factors; terms associated with the (b) impact of malnutrition on cardiovascular risk and complications in CKD patients; and terms related to the (c) dietary protein intake and malnutrition in CKD.

**Conclusions:**

This study is the first of its kind to analyze CKD and malnutrition research using data from Scopus for visualization and network mapping. Recent trends indicate an increasing focus on protein-energy wasting/malnutrition in hemodialysis patients and predicting factors, dietary protein intake, and malnutrition in CKD. These topics have gained significant attention and reflect the latest scientific advances. Intervention studies are crucial to examining diet therapy's impact on patients with stages 1 to 5 CKD. We hope this study will offer researchers, dietitians and nephrologists valuable information.

## Background

Chronic kidney disease (CKD), frequently referred to as kidney failure, is a potentially fatal condition that has emerged as the primary global cause of mortality [1, 2]. The condition is distinguished by a progressive decline in renal function, resulting in potentially hazardous fluid, salt, and mineral disruptions [3]. CKD is a complex and diverse condition characterized by various etiological factors that impact the morphology and physiological capabilities of the kidneys. These factors encompass diabetes, hypertension, obesity, cardiovascular disease, and aging [4, 5]. Moreover, CKD is recognized as a clinically insidious condition, particularly during its early stages, characterized by limited manifestations and indications, resulting in the underdiagnosis of kidney disease until it has advanced [6–8].

The chronicity criterion for diagnosing CKD requires repeated measurements of low estimated glomerular filtration rate (eGFR) or elevated urine albumin over at least 90 days. CKD stages 3–5 are typically determined by eGFR, while studies combining albuminuria and decreased eGFR report stages 1–5 [6, 9]. The prevalence of CKD varies in developing countries but generally affects more than 10% of the population, with more than 800 million individuals affected worldwide [6]. Deaths associated with CKD have increased in the past two decades due to diabetes and obesity [6, 10].

Early detection and treatment might help prevent kidney disease from progressing to the last stage of kidney failure, especially in low- and middle-income countries where CKD represents a heavy burden, as they are the least equipped countries to deal with its consequences. Treatments for CKD can delay progression, stop the onset, reduce complications from GFR decline, lower the risk of cardiovascular disease, increase survival rates, and improve the quality of life [11].

Nutrition and nutritional status are essential for health, survival, quality of life, and many other aspects of daily living. On the other hand, malnutrition is a serious complication of kidney disease due to disease burden and inflammation linked to higher morbidity, death, and expenses. CKD patients are at risk of metabolic and nutritional problems that increase energy expenditure, encourage protein catabolism, reduce appetite and food intake, and result in a condition known as protein energy wasting [12, 13].

The Global Leadership Initiative on Malnutrition (GLIM) proposed a combination of three phenotypic criteria (unintentional weight loss, low body mass index (BMI), and reduced muscle mass) and two etiologic criteria (reduced food intake or assimilation; disease burden or inflammatory condition) as a consensus, thorough, and workable procedure to diagnose malnutrition in clinical and research settings. When the patient meets at least one combined phenotypic and etiologic criterion, malnutrition is diagnosed [14]. Most patients with severe CKD meet both etiological criteria developed by the GLIM criteria because they frequently experience systemic inflammation and anorexia caused by the retention of uremic toxins. The prevalence of malnutrition diagnosed by GLIM was between 12 and 54.3%, depending on the stage of CKD, the population, and the combination of criteria used [15]. Although GLIM has been validated against various semi-gold standard parameters and represents a significant advancement in the diagnosis of malnutrition across various patient categories and clinical settings [16], studies are still relatively new and scarce. Further research is needed on patients at different stages of CKD to fully comprehend its applicability and value in clinical practice [15]. This is because muscle mass and the three phenotypic criteria suggested by GLIM can be influenced in patients with fluid retention at an advanced stage of CKD, leading to an underestimation of malnutrition.

Body weight and BMI are also influenced, as many kidney failure patients suffer from obese sarcopenia that may be related to inflammation [17]. As a result, several studies recommended implementing GLIM as a complement to the diversity of methods and tools already used for the diagnosis of malnutrition in CKD, such as subjective global assessment (SGA) and malnutrition-inflammation score (MIS), which apply a set of composite scores to diagnose malnutrition [14, 15, 18].

Many pharmacological treatments are often utilized to reduce the occurrence or seriousness of comorbidities in CKD. However, these treatments have substantial side effects and high costs [19]. In contrast, nutrition therapy has become the first-line treatment option in multiple therapeutic approaches, and nutritional requirements fluctuate as CKD progresses to protect bones and blood vessels; therefore, an eating plan is crucial as part of a nutritional intervention regimen [20]. Therefore, this study provides information on the current state of research on malnutrition CKD through a bibliographic analysis. Bibliometrics is a useful method to obtain an overview of malnutrition, and CKD has evolved to the point where it is currently considered a high-quality, reliable, and informative tool. This analysis may not provide strong evidence relevant to the research question, but it does provide insight into the problem and solutions discussed globally in an area of interest and consideration. The intellectual structure of research, knowledge dissemination, emerging literature, and trends over time can help identify problems in the medical field, as well as the gap presented between theory and practice [21]. Consequently, a bibliometric analysis will be conducted to increase the efforts to make for better diagnosis and treatment due to the large number of affected people and the serious negative effects. This study highlights the importance of studying malnutrition in CKD, identifies the research landscape in this area, and emphasizes the need for continued efforts to address malnutrition as a significant factor in the progression of CKD and associated complications. The findings will contribute to the knowledge base and provide information on future research directions and clinical interventions aimed at improving outcomes for people with CKD.

## Methods

### Study design

We performed a web-based bibliometric investigation utilizing the Scopus database provided by Elsevier and can be accessed at http://www.scopus.com. The authors have reviewed the PRISMA 2020 Checklist, and the manuscript was composed and revised in accordance with the PRISMA 2020 Checklist [22].

### Database

Scopus, the most reputable scientific research database, provided our data. Scopus provides the most important data sources for bibliometric analysis and lets scholars create thorough search queries utilizing various inquiry methods [23–28]. It also has a more consistent and standardized record in the study of literature in multiple disciplines [29–36]. Bibliometric analysis involves quantitative and qualitative study of papers from SciVerse, Scopus, or the Web of Knowledge. Gray literature is excluded from bibliometric analysis. Researchers choose SciVerse Scopus because database selection is critical. Scopus, founded by Elsevier, showed healthy bibliometric growth. It has advantages over Web of Science, Medline and Google Scholar in several researches. (1) Scopus contains vast scientific literature from peer-reviewed journals, conference proceedings, book series, trade publications, and patents. Its broad reach makes it useful for analyzing research trends across disciplines. (2) Scopus carefully selects its sources, resulting in high-quality data. Scopus also has content specialists who evaluate data quality and update it often to maintain accuracy. (3) Scopus allows advanced searches by author, publication, affiliation, keyword, and citation. (4) Scopus provides citation measures, including citation counts, h-index, and cocitation analysis, which are utilized in bibliometric studies to evaluate research papers and authors. (5) Scopus integrates with data visualization and research analytics platforms, making bibliometric data analysis and presentation easy. (6) Scopus is a complete bibliometric repository because it covers most Web of Science, MEDLINE and EMBASE journals. Scopus, the most popular and respected research database, contains papers from top journals with global influence. The current study used the same bibliometric indicators as prior investigations [37, 38].

All information was taken from Scopus on June 2, 2023, to avoid any potential bias brought on by the periodic database updates of Scopus.

### Search strategies

The relevant terms related to CKD and malnutrition were identified using PubMed Medical Subject Headings (MeSH) and by consulting pertinent publications on this subject [39–41]. These identified terms were subsequently incorporated into the Scopus Engine. Each selected "keyword" was utilized as an entry for the "Article Title, Article Abstract, and Author Keywords" fields.

The study focused on terms such as 'Malnutrition' or 'Nutritional Deficienc*' or 'Undernutrition' or 'Malnourishment', which directly addressed the issue of malnutrition itself rather than other associated terminologies such as specific micronutrient deficiencies. Similarly, the study employed keywords such as 'Chronic Renal Insufficienc*' or 'Chronic Kidney Disease*' or 'Chronic Kidney Disease*' or 'Chronic Kidney injury' or 'CKD' or 'Chronic Renal Disease*' or 'Chronic Kidney Insufficienc*' or 'Chronic Renal Injury', which specifically pertained to 'Chronic Kidney Disease' itself rather than other related terms such as specific kidney diseases.

The terms were used to search titles, abstracts and author keywords to optimize document retrieval. Asterisks and quotation marks were utilized in the search string to simplify and enhance the accuracy of the search. The search strategy focused exclusively on publications published in peer-reviewed scientific journals, excluding erratum and retraction documents.

This study used data from Scopus published between January 1, 2003, and December 31, 2022. The last two decades were chosen because they are assumed to provide a better picture of the pattern of publications and citations received. Additionally, this time range allows for comparison of earlier and more recent periods to identify shifts, growth, or declines in research output and impact. By limiting the study to the years 2003–2022, the researchers were able to capture the most recent trends in the research on malnutrition in CKD. In addition, this time range encompasses a significant and crucial phase of research development in the field. For example, research on malnutrition in patients with chronic kidney disease gained notable attention and development after 2000 despite SGA being first introduced in CKD in 1987 [42, 43].

### Validation of the search strategy

A random sample of 50 articles was chosen from the retrieved literature. Only articles with even numbers (15, 30, 45, 60, etc.) were included in the sample until the end of the retrieved document list. This selection was made to ensure the results' validity and prevent false-positive results. The top 50 articles cited in the retrieved dataset were also reviewed to further validate the accuracy of the results. It is important to mention that the validation approach used in this study was previously employed by Sweileh et al. [44–46] and Zyoud et al. [34, 47].

### Data export and management

To examine bibliometric indicators, we transferred the collected data to Microsoft Excel for analysis and organization. We obtained significant bibliometric data on research publications on malnutrition and chronic kidney disease, including the annual article count, types of documents, countries/regions involved, institutions, funding agencies, journals along with their impact factors (IF), citation patterns, and the h-index. Our analysis of these findings was carried out using descriptive statistics.

### Visualization analysis

The study focuses on analyzing the clustering and co-occurrence of terms found in the titles and abstracts of publications that address the topics of malnutrition and CKD. The research also explores the visualization of these findings through overlay visualization and investigates the collaboration network between multiple countries. VOSviewer software version 1.6.19 was utilized to conduct this analysis [48, 49]. The size of the node and the word on the map represented the frequency of co-occurrence, and the link between nodes indicated their relationship. Nodes that shared the same color were part of the same cluster. We then divided the keywords by specific colors based on the average number of times they appeared in all publications. A visualization map was created that overlays terms from the titles and abstracts of publications related to malnutrition and CKD. Each keyword was assigned a different color based on its average appearance time in the overlay visualization map. The color blue was used to represent keywords that appeared earlier in the time course compared to those in yellow and green.

## Results

### General characteristics of the retrieved articles

Extensive research conducted on a global scale between 2003 and 2022 has resulted in an impressive compilation of 50,588 documents focused on CKD, as indicated by titles and author keywords. Within this vast collection of scholarly publications, a notable subset of 823 articles was dedicated to investigating the relationship between CKD and malnutrition. Further analysis reveals that this body of work consists of 565 articles (68.65%), 221 reviews (26.85%), and 37 miscellaneous entries (4.50%), which encompass letters and editorials.

### Evolution and growth of publications

**Fig. 1:**
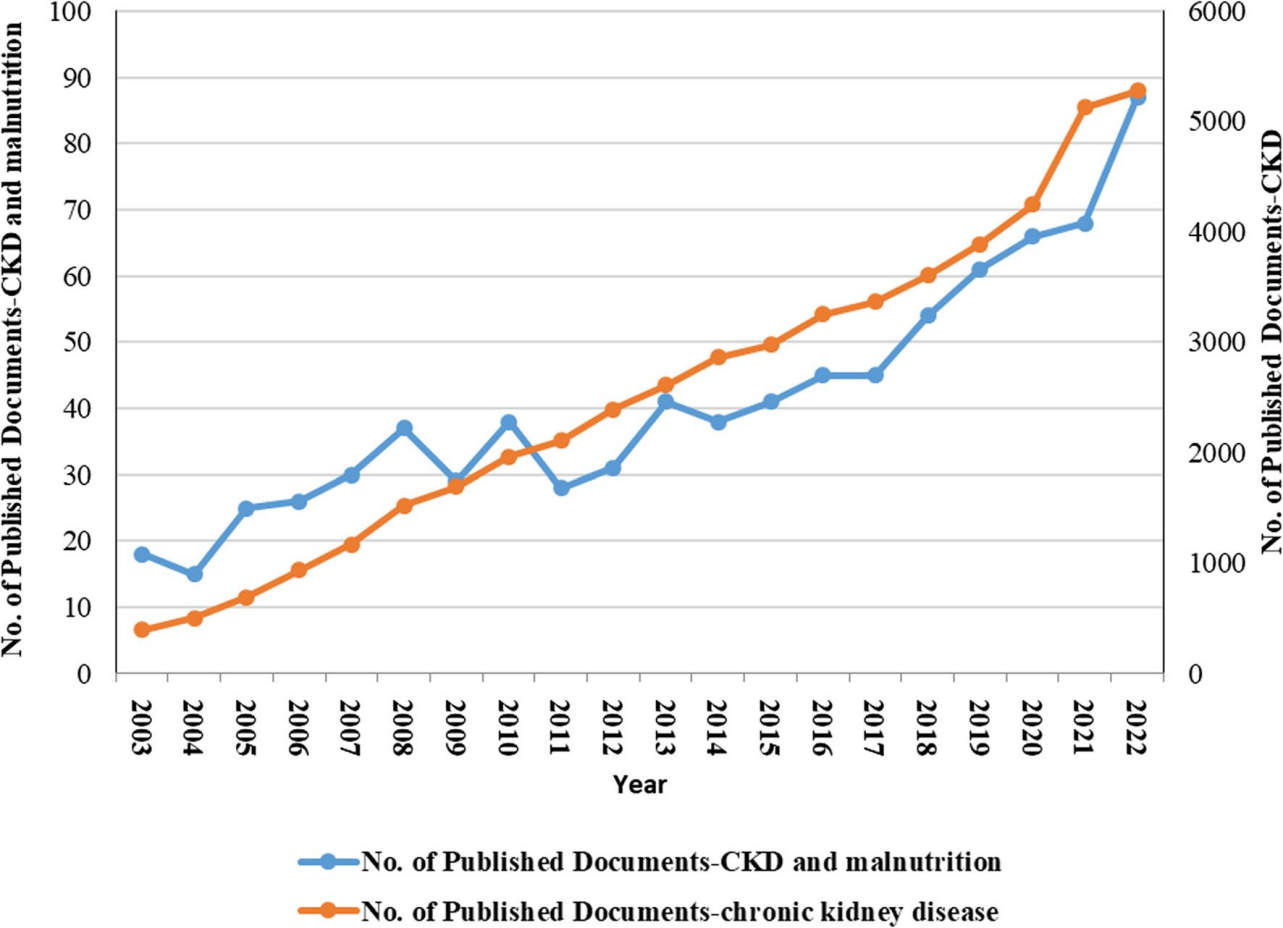
The annual number of publications related to malnutrition and chronic kidney disease from 2003 to 2022

Figure 1 illustrates the trends in Scopus publications concerning CKD and malnutrition over the last two decades. A highly significant and robust positive correlation (*r* = 0.946, *P* < 0.001) was observed between publication productivity in various fields regarding CKD and productivity specifically focused on CKD and malnutrition. Initially, the number of publications experienced a slight increase from 2003 to 2017, with an annual average of fewer than 32.47 articles. However, from 2017, there was a rapid growth in articles specifically addressing CKD and malnutrition, averaging more than 67.2 publications per year. This upward trajectory peaked in 2022, reaching a total of 87 publications.

### Top ten active countries

**Fig. 2:**
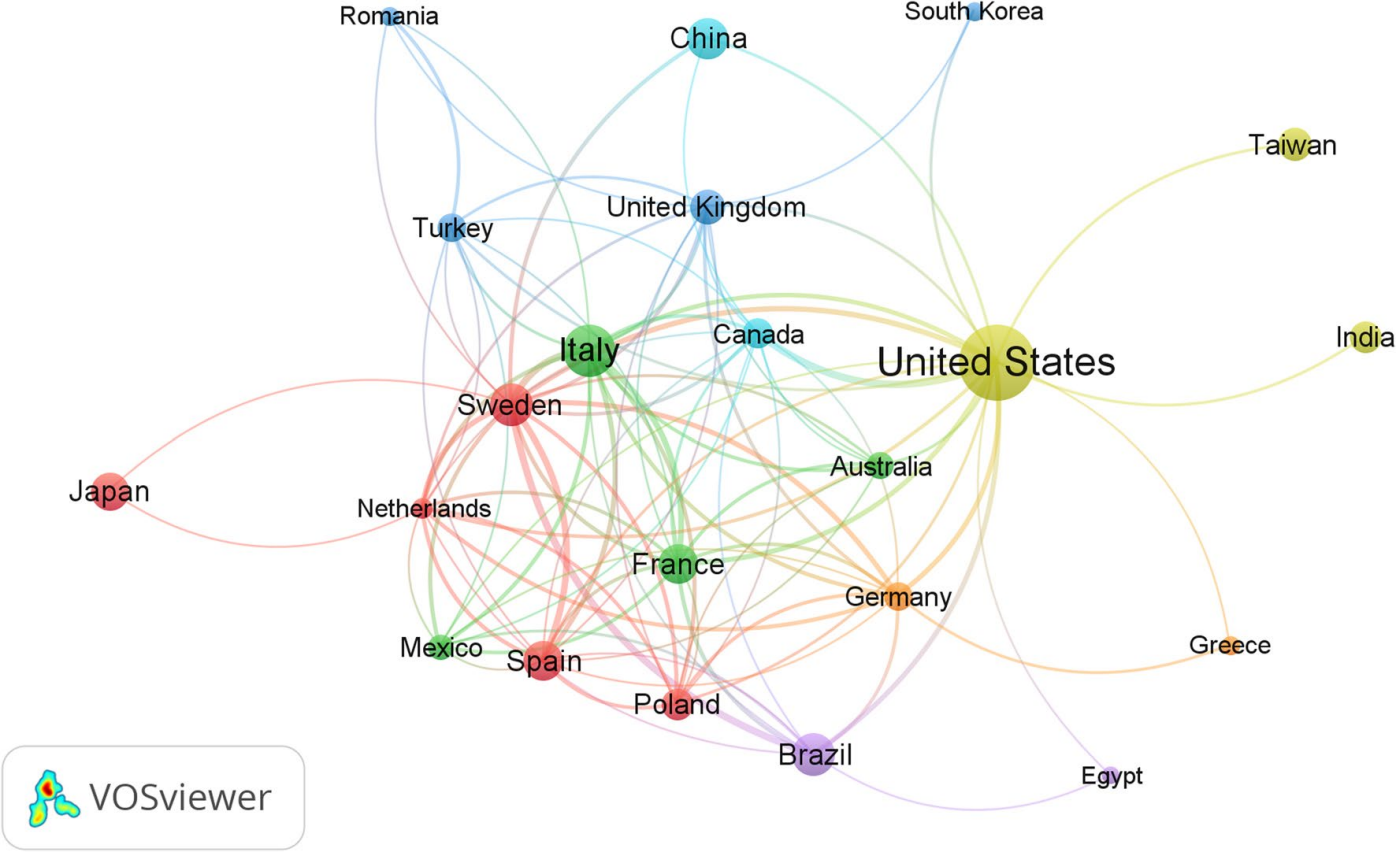
A network of countries and regions that collaborate to study malnutrition and chronic kidney disease. The cooperation map revealed that 22 countries and regions had produced at least ten publications on the subject

Researchers from 71 countries signed all included publications related to CKD and malnutrition. Table 1 shows that the USA was found to be the most productive country (*n* = 173; 21.02%), the Republic of Italy was found to be the second most productive country with (*n* = 83; 10.09%) publications, and Sweden (*n* = 56; 6.8%), Brazil (*n* = 54; 6.56%), China (*n* = 51; 6.20) and Spain (*n* = 47; 5.71%) followed. Figure 2 represents a network of collaborating studies on malnutrition and chronic kidney disease in 22 different countries and regions, each contributing at least 10 publications. The level of cross-country collaboration can be seen in the thickness of the connecting lines and the size of the nodes, with the USA showing the strongest alliances.

**Table 1:** Top ten countries most productive in terms of relevant articles related to malnutrition and chronic kidney disease between 2003 and 2022 (*n* = 72)

Ranking	Country	No. of documents	%
1st	United States	173	21.02
2nd	Italy	83	10.09
3rd	Sweden	56	6.80
4th	Brazil	54	6.56
5th	China	51	6.20
6th	Spain	47	5.71
7th	France	45	5.47
8th	Japan	45	5.47
9th	UK	36	4.37
10th	Taiwan	34	4.13

### Top ten active institutions

According to the number of publications, Table 2 presents the ten most productive institutions researching malnutrition and CKD. The institution with the highest scientific production is *Karolinska Institutet* in Sweden, accounting for 48 publications (5.83%). The *David Geffen School of Medicine at UCLA* in the USA is closely followed, with 27 publications (3.28%). The *University of California, Los Angeles* (USA), ranks third in production, with 26 publications (3.16%).

**Table 2:** Top ten productive institutes in research related to malnutrition and chronic kidney disease from 2003 to 2022

Ranking	Institute	Country	No. of documents	%
1st	*Karolinska Institutet*	Sweden	48	5.83
2nd	*David Geffen School of Medicine at UCLA*	USA	27	3.28
3rd	*University of California, Los Angeles*	USA	26	3.16
4th	*The Lundquist Institute*	USA	24	2.92
5th	*Harbor-UCLA Medical Center*	USA	23	2.79
6th	*Karolinska Universitetssjukhuset*	Sweden	21	2.55
7th	*VA Medical Center*	USA	16	1.94
7th	*Università degli Studi di Milano*	Italy	16	1.94
9th	*Universidade Federal de São Paulo*	Brazil	15	1.82
10th	*Ospedale Maggiore Policlinico Milano*	Italy	14	1.70

### Top ten active funding agencies

Table 3 shows the top ten active funding agencies. The *National Institute of Diabetes and Digestive and Kidney Diseases* (*n* = 46, 5.59%), the *National Institutes of Health* (*n* = 23, 2.79%) and the *National Center for Research Resources* (*n* = 15, 1.82%) were found to be the most active funding agencies in this field in the United States, followed by the *National Natural Science Foundation of China* (China) with 13 publications (1.58%), the *National Center for Advancing Translational Sciences* (USA) with 11 publications (1.34%), and *Coordenação de Aperfeiçoamento de Pessoal de Nvel Superior* (Brazil), *Vetenskapsrådet* (Sweden) with 9 publications (1.09%).

**Table 3:** Top 10 productive funding agencies in research related to malnutrition and chronic kidney disease from 2003 to 2022

Ranking^a^	Funding agency	Country	No. of documents	%
1st	*National Institute of Diabetes and Digestive and Kidney Diseases*	USA	46	5.59
2nd	*National Institutes of Health*	USA	23	2.79
3rd	*National Center for Research Resources*	USA	15	1.82
4th	*National Natural Science Foundation of China*	China	13	1.58
5th	*National Center for Advancing Translational Sciences*	USA	11	1.34
6th	*Coordenação de Aperfeiçoamento de Pessoal de Nível Superior*	Brazil	9	1.09
7th	*Vetenskapsrådet*	Swedish	9	1.09
7th	*National Heart, Lung, and Blood Institute*	USA	6	0.73
9th	*Amgen*	USA	5	0.61
9th	*Baxter Healthcare Corporation*	USA	5	0.61
9th	*Consejo Nacional de Ciencia y Tecnología*	Mexico	5	0.61
9th	*Ministry of Science and Technology, Taiwan*	Taiwan	5	0.61

### Top ten active journals

Table 4 presents the top 10 research journals/source titles that have published extensive articles on malnutrition and CKD and their impact factors (IFs) for 2022. Among these journals, nutrients demonstrated the highest productivity, contributing 47 documents, which accounted for 4.71% of the total publications. The *Journal of Renal Nutrition* (*n* = 28, 3.40%), *Pediatric Nephrology* (*n* = 26, 3.16%), *American Journal of Kidney Diseases* (*n* = 22, 2.67%), and *Nephrology Dialysis Transplantation* (*n* = 20, 2.43%)**.**

**Table 4:** Top ten productive journals/source titles in research related to malnutrition and chronic kidney disease from 2003 to 2022

Ranking^a^	Journal/source title	No. of documents	%	IF ^b^
1st	*Nutrients*	47	5.71	5.9
2nd	*Journal of Renal Nutrition*	28	3.40	3.2
3rd	*Pediatric Nephrology*	26	3.16	3.0
4th	*American Journal of Kidney Diseases*	22	2.67	13.2
5th	*Nephrology Dialysis Transplantation*	20	2.43	6.1
6th	*Nefrologia*	16	1.94	2.6
7th	*Kidney International*	14	1.70	19.6
7th	*Nutricion Hospitalaria*	14	1.70	1.2
9th	*Clinical Journal of the American Society of Nephrology*	13	1.58	9.8
9th	*International Urology and Nephrology*	13	1.58	2.0

^a^ When specific journals are given the same ranking number, a gap is left in the next ranking number

^b^ Impact factors (IF) based on Clarivate Analytics Journal Citation Reports (JCR) 2022

### Citation patterns

According to the analysis of citations, the retrieved articles had an average of 28.24 citations. They achieved an h-index of 75, accumulating a total of 23,245 citations. The range of citations for these articles ranged from 0 to 1356. Of the total, 120 articles received no citations, while 48 garnered 100 or more citations. There was a wide range in the total number of citations for these publications, from 207 to 1356 (Table 5) [16, 50–58]. The ten most cited publications in research related to malnutrition and CKD from 2003 to 2022 are in 2008 by Fouque et al. [51] titled “A proposed nomenclature and diagnostic criteria for protein-energy wasting in acute and chronic kidney disease" followed by “Reverse epidemiology of conventional cardiovascular risk factors in patients with chronic heart failure” in 2004 by Kalantar-Zadeh et al. [54]. The third most cited article was related to Carrero et al. [16] in 2004 “Etiology of the Protein-Energy Wasting Syndrome in Chronic Kidney Disease: A Consensus Statement from the International Society of Renal Nutrition and Metabolism (ISRNM)”.

**Table 5:** The 10 most cited publications in research related to malnutrition and chronic kidney disease from 2003 to 2022

Authors	Title	Year	Source title	Cited by
Fouque et al. [51]	“A proposed nomenclature and diagnostic criteria for protein-energy wasting in acute and chronic kidney disease”	2008	*Kidney International*	1356
Kalantar-Zadeh et al. [54]	“Reverse epidemiology of conventional cardiovascular risk factors in patients with chronic heart failure”	2004	*Journal of the American College of Cardiology*	544
Carrero et al. [16]	“Etiology of the Protein-Energy Wasting Syndrome in Chronic Kidney Disease: A Consensus Statement From the International Society of Renal Nutrition and Metabolism (ISRNM)”	2013	*Journal of Renal Nutrition*	527
Ikizler et al. [53]	“Prevention and treatment of protein energy wasting in chronic kidney disease patients: A consensus statement by the International Society of Renal Nutrition and Metabolism”	2013	*Kidney International*	437
Wong et al. [57]	“Expansion of urease- and uricase-containing, indole- and p-cresol-forming and contraction of short-chain fatty acid-producing intestinal microbiota in ESRD”	2014	*American Journal of Nephrology*	405
Akchurin and Kaskel [50]	“Update on inflammation in chronic kidney disease”	2015	*Blood Purification*	354
McIntyre et al. [55]	“Circulating endotoxemia: A novel factor in systemic inflammation and cardiovascular disease in chronic kidney disease”	2011	*Clinical Journal of the American Society of Nephrology*	350
Rambod et al. [56]	“Association of Malnutrition-Inflammation Score With Quality of Life and Mortality in Hemodialysis Patients: A 5-Year Prospective Cohort Study”	2009	*American Journal of Kidney Diseases*	262
Workeneh and Mitch [58]	“Review of muscle wasting associated with chronic kidney disease”	2010	*American Journal of Clinical Nutrition*	210
Friedman et al. [52]	“Demographics and trends in overweight and obesity in patients at time of kidney transplantation”	2003	*American Journal of Kidney Diseases*	207

### Analysis of co-occurrence

**Fig. 3:**
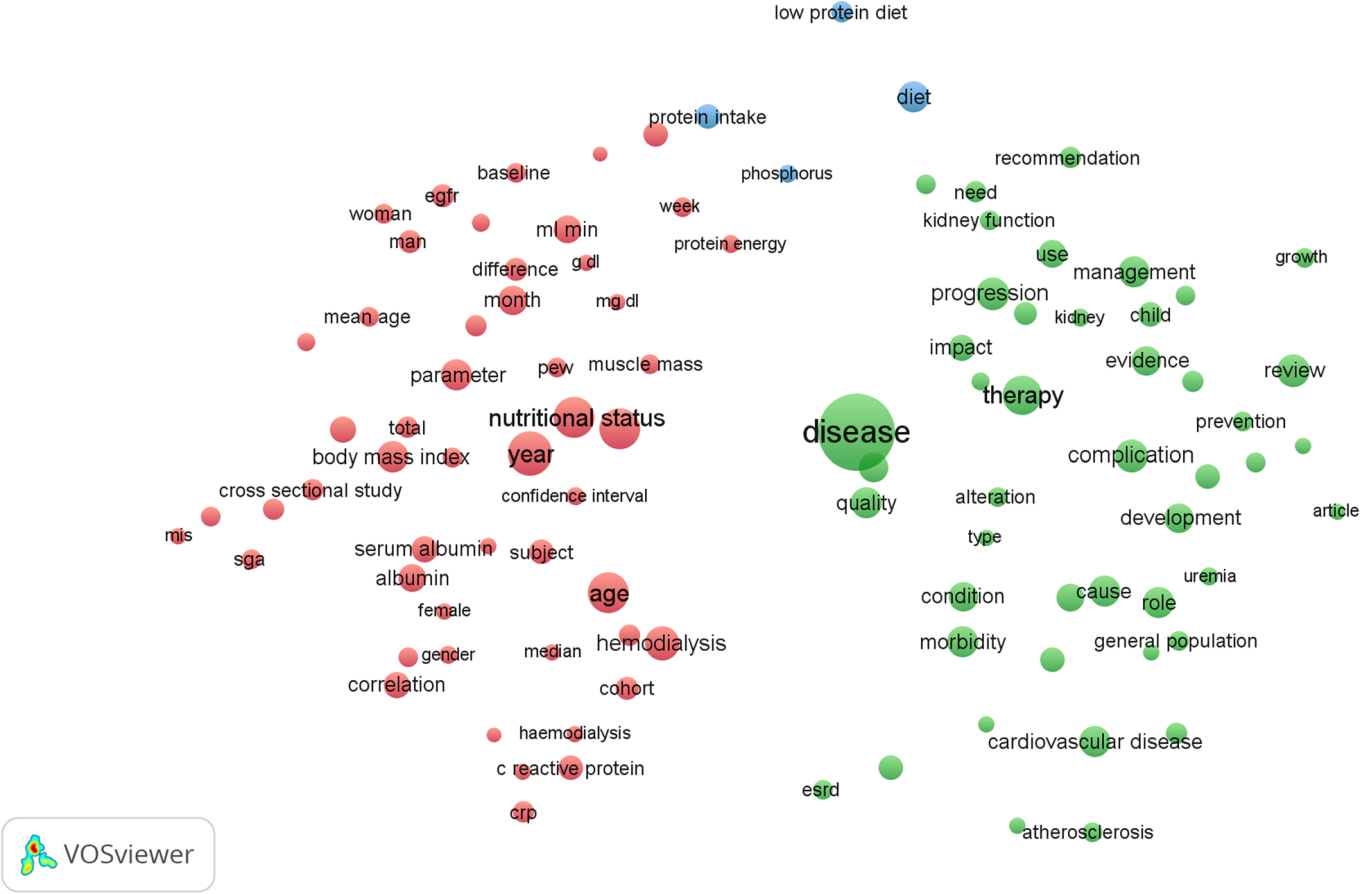
The clustering and co-occurrence of terms in the title and abstract fields of publications related to malnutrition and chronic kidney disease. A map of the clusters revealed 169 terms that occurred at least 30 times, which were categorized into three separate clusters. The size of the node and the word on the map represented the frequency of co-occurrence, and the link between nodes indicated their relationship. Nodes that shared the same color were part of the same cluster

**Fig. 4:**
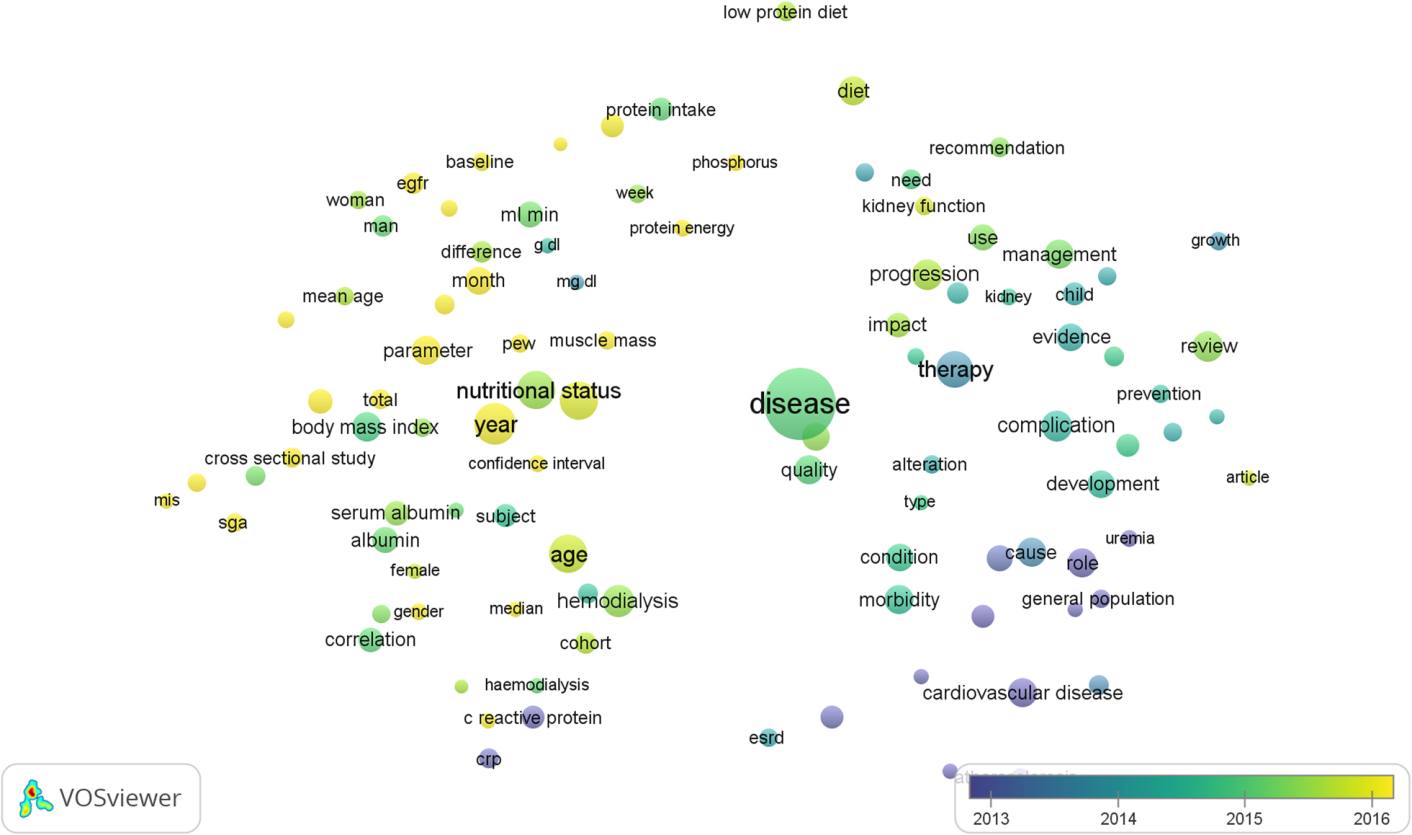
A visualization map has been created that overlays terms from the titles and abstracts of publications related to malnutrition and chronic kidney disease. Each keyword was assigned a different color based on its average appearance time in the overlay visualization map. Blue was used to represent keywords that appeared earlier in the time course compared to those in yellow

To identify the terms most frequently mentioned in the collected publications, VOSviewer was used. Of the 16,251 terms in this field, 196 met the criteria of appearing at least 30 times. These terms were categorized into three distinct clusters, each represented by a different color. The prominent topics within malnutrition and CKD were represented by three clusters: green, blue, and red. Cluster number 1 (red) encompassed terms related to the issue of “malnutrition in hemodialysis patients and predicting factors.” Cluster number 2 (green) comprised terms associated with the “impact of malnutrition on cardiovascular risk and complications in patients with CKD.” Cluster number 3 (blue) included terms related to the “dietary protein intake and malnutrition in CKD (see Fig. 3). VOSviewer color-coded the terms based on their average appearance in the 823 relevant publications (see Fig. 4). Before 2015, the majority of studies focused on the “impact of malnutrition on cardiovascular risk and complications in CKD patients” (indicated by the presence of blue terms). However, recent trends suggest that “malnutrition in hemodialysis patients and predicting factors” and “dietary protein intake and malnutrition in CKD” (indicated by green to yellow terms) will receive greater attention in the future.

### Future research direction analysis

In Fig. 4, VOSviewer used different colors to represent each term in all publications related to malnutrition and CKD based on the average appearance in the overlay visualization map. In contrast to the keywords in yellow and green, those that came earlier in the time course were represented by the color blue. Studies before 2014 focused primarily on the “impact of malnutrition on cardiovascular risk and complications in CKD patients”. However, the most recent research trends discovered that “malnutrition in hemodialysis patients and predicting factors” and “dietary protein intake and malnutrition in CKD” are the most recent.

## Discussion

In this study, a bibliometric analysis of malnutrition and CKD studies was performed using the Scopus global database with the help of VOSviewer software to find hotspots and emerging trends. We focus specifically on studies released between 2003 and 2022. A notable subset of 823 articles investigated the relationship between CKD and malnutrition. Several studies have shown a deadly broad area between kidney and cardiovascular disease that requires urgent and focused attention [59–61]. The number of publications experienced a slight increase from 2003 to 2017. However, from 2017, there was an upward trajectory of rapid growth in the number of published articles specifically addressing CKD and malnutrition that peaked in 2022 and that we expect to continue in the predictable future.

Several components can clarify the increase in the yield investigated. In general, nutrition has gained more importance in preventing and treating chronic diseases. It has attracted the attention of a multidisciplinary medical team since nutrition management using a multidisciplinary approach improves patients’ nutritional status during medical treatments [62]. Additionally, research methods can help researchers study nutrition therapy's role in slowing CKD progression [63], aligning with increased research funding strategies [64]. These findings provide insight into the global problem of malnutrition and CKD research and help to understand risks, prevention, and treatment.

The United States administration has adequate resources with strong collaboration and partnerships, making it the worldwide leader in food and nutritional research [32, 65, 66]. This article found that the USA is the most productive country regarding malnutrition and CKD.

The “impact of malnutrition on cardiovascular risk and complications in CKD” has emerged as one of the hot topics to focus on in our study. CVD is the most common type of heart disease and the leading cause of death worldwide, accounting for 45–50% of deaths among end-stage dialysis patients [67]. CVD is a reduction in blood flow to the heart due to atherosclerosis, resulting in plaque accumulation in the heart arteries [68]. Most patients with CKD have a significantly high incidence of cardiovascular diseases and comorbidities, such as malnutrition and inflammation, which can result in poor prognosis and premature cardiovascular death [59]. The risk of heart failure in people with CKD is specifically linked to malnutrition [69]. Anemia and malnutrition can be the cause or effect of frequent inflammation in patients with CKD, which is a higher risk factor than dyslipidemia [68, 70].

In addition, patients with CKD experience uremia-specific factors such as anemia, hyperparathyroidism, carnitine deficiency, hyperhomocysteinemia, and low levels of vitamin C, which contribute further to the development of cardiovascular disease. Furthermore, hypertension, dyslipidemia, diabetes, and obesity, commonly known as causes of CKD and CVD in the general population, increase the risk of cardiovascular disease in patients with CKD. The existence and severity of ischemia observed during stress echocardiography were identified as an autonomous indicator of mortality among CKD patients, and this association persisted even after the patients received a kidney transplant [71].

In contrast, it has not been demonstrated that adjusting hyperhomocysteinemia using pyridoxine, vitamin B12, and folic acid supplements effectively lowers cardiovascular risk in kidney patients [71]. Another interesting result on traditional CV risk factors indicated that a higher body mass index is associated with reduced cardiovascular risk than a low body mass index once patients are on dialysis [72], which could shed light on the importance of healthy weight gain for chronic diseases to prevent malnutrition. Therefore, intervention studies are still lacking on the effects of malnutrition and inflammation and are not precise, reliable and consistent for widespread practice. Furthermore, it is possible to improve the nutrition care process and reduce malnutrition, particularly during the initial phases of CKD, through meticulous evaluation and therapy that includes a collaborative group of experts in anemia, nursing, nephrology and dietetics [73]. As a result, future research should include experimental studies in the early stages of CKD to obtain maximum patient benefits and generalize updated effective guidelines for best practices in healthcare facilities.

‘Malnutrition in hemodialysis patients and predicting factors’ as a theme was among the main hot topics in the current study. A significant number of individuals with advanced CKD exhibit noticeable clinical signs of protein energy malnutrition and wasting. These conditions result from decreased food consumption due to anorexia, nausea, and vomiting caused by uremic toxicity, negatively affecting both muscle stem cells and skeletal muscle. Malnutrition is also associated with infections, disturbances in hormones and body metabolism, and increased mortality from cardiovascular diseases in CKD patients [74]. However, numerous reports indicate that the incidence of protein-energy malnutrition remains elevated in people undergoing hemodialysis, ranging from 23 to 76%. This prevalence is influenced by age, dialysis treatment effectiveness and comorbidities [75, 76].

The complex mechanisms that lead to muscle protein and fat loss can involve multiple abnormalities that stimulate protein breakdown or decrease protein synthesis [74]. Clinical factors linked to malnutrition included advanced age, cardiovascular disease and diabetes mellitus. Serum albumin was affected not only by nutritional status and protein consumption but also independently by age, serum C-reactive protein (SCRP), and sex. Plasma levels of insulin-like growth factor 1 (IGF-1) also indicated the existence and severity of malnutrition. They seemed to be more strongly correlated with indicators of somatic protein mass compared to serum albumin. Elevated SCRP, which primarily indicates the presence of infection or inflammation, is more prevalent among older patients than younger patients [77].

Medical nutrition therapy (MNT) was established as a legal term to allow patients to receive personalized nutrition counseling for ‘pre-end stage renal disease’ from qualified dietitians to slow or prevent disease progression. Continued assessment of nutritional status is important and should start as early as possible to minimize uremic toxicity, correct several metabolic abnormalities and symptoms, and prevent waste and malnutrition. This can be achieved by providing adequate calories to prevent catabolism of body tissues during the patient’s hospital stay. Therefore, many studies related to nutrition care highlight inadequate nutritionist and lack of consistency in hospital access to nutrition care and the negative consequences on patients’ hospital stay and cost savings [73, 78].

Another key topic of discussion in the current investigation was “dietary protein intake and malnutrition in CKD”. Several studies in HD patients have established that hypoalbuminemia reflects the presence of malnutrition and is considered a strong independent predictor of cardiovascular mortality. Research has also demonstrated a correlation between indications of malnutrition, especially reduced serum albumin levels, and elevated morbidity and mortality rates [79, 80]. However, studies have revealed that although serum albumin is a poor nutritional indicator in CKD, it is still the most commonly used nutritional marker for nutritional status [81, 82].

The protein recommendations are designed to offset the reductions caused by dialysis, irregularities in protein metabolism, alterations in albumin turnover, heightened breakdown of amino acids, inflammation, and infection. The recommended protein intake for CKD patients varies depending on the treatment methods, the types of protein, grams of protein per kilogram of standard body weight, and the stage of the disease in infected patients [20]. Thus, numerous intervention studies should highlight the importance of alternative measurement methods to assess nutritional status in hospitalized patients and walk-in facilities in medical centers.

## Strength and limitations

This study breaks new ground by offering an extensive overview of publications concerning CKD and malnutrition over the last two decades. As a result, researchers might use visual analysis to improve the research areas of malnutrition and development trends in malnutrition and CKD. While relying on a single database could be considered a limitation, the decision to utilize a large and comprehensive database for visualizing and mapping literature remains valid and defensible. It is important to acknowledge the potential for omitting certain articles. Therefore, the findings of this study should be interpreted in light of this potential limitation, which could impact the rankings of countries, institutions, funding agencies, and journals. Our study's objective was not to examine the difference between malnutrition risk and nutritional risk; despite the consensus around defining malnutrition, there is still no distinction between the definitions of malnutrition risk and the general name of the nutritional risk that is characterized by decreased food intake, both in terms of quantity and quality, which can lead to malnutrition over time [83]. Consequently, our study focused on terms such as ‘Malnutrition’ or ‘Nutritional Deficienc*’ or ‘Undernutrition’ or 'Malnourishment', which directly addressed the issue of malnutrition itself rather than other associated terminologies such as specific micronutrient deficiencies. In addition, because citation searches are “time dependent,” some older articles may dominate the list of most recent top-cited list articles. Finally, the scope of the present review was limited to the title and abstract search and included only those malnutrition and CKD phrases. As a result, this scan may have missed certain articles that used other related terms as keywords or contained those terms anywhere in the publication’s text.

## Conclusions

To date, no comprehensive evaluation of the evidence has been carried out regarding malnutrition and CKD, and our search did not reveal any ongoing bibliometric studies on the topic. The study illuminates the correlation between malnutrition and CKD, highlighting the impact of malnutrition on cardiovascular risk and complications in patients with CKD. People with CKD often suffer from malnutrition. It can exacerbate the disease and cause additional health problems. Since 2017, the number of studies on malnutrition in CKD has increased, indicating a developing interest in this field. The USA, Italy, and Sweden are in the lead in this bibliometric investigation. Malnutrition must be diagnosed and treated promptly in patients with CKD to prevent disease progression. In managing CKD and its complications, nutrition therapy and diet interventions are crucial. The manuscript emphasizes the importance of nutritional therapy as a cost-effective and side-effect-free alternative to pharmaceutical treatments. Furthermore, it indicates a shift in research focus toward investigating malnutrition in hemodialysis patients, identifying factors associated with malnutrition, and examining the influence of malnutrition on cardiovascular risk among patients with CKD. These emerging trends highlight the need for additional research to improve our understanding and management of malnutrition in the context of CKD.

### Clinical perspectives in the future

Research on CKD and malnutrition has many implications.

- Investigations of the nutrition care process of patients with chronic kidney disease from the initial phases include collaborating expert groups to minimize malnutrition and other comorbidities.
- Studies related to nutrition care highlight the inadequate nutritionist and lack of consistency in access to nutrition care in hospitals and the negative consequences on patients’ hospital stay and cost savings.
- Experimental studies should be included in the early stages of CKD in future research to obtain the maximum benefits for patients and generalize updated effective guidelines for best practices in healthcare facilities. Continued assessment of nutritional status is important to prevent waste and malnutrition.
- In the future, nutrition and diet therapy interventions for chronic kidney diseases should be studied. These diets must be tested for their effectiveness and long-term sustainability in prevention and treatment. In addition, nutrition that aligns with drug therapy and invasive therapy in CKD can improve and decrease the side effects of renal replacement therapy.
- The recent shift in research focus toward malnutrition and dietary protein intake in CKD indicates the need for additional studies on this subject. Future research should investigate the underlying mechanisms and risk factors contributing to malnutrition in patients with CKD on hemodialysis and highlight the importance of alternative measurement methods to assess nutritional status in hospitalized patients and walk-in facilities in medical centers.
- Interactive collaboration and knowledge sharing can improve research on malnutrition and CKD. Collaborations between researchers from different countries and disciplines can provide a broader perspective and contribute to developing strong evidence-based guidelines to minimize the negative effects on patients with chronic diseases.

## Data Availability

All data generated or analyzed during this study are included in this published article. In addition, other datasets used during the current study are available from the corresponding authors upon reasonable request.

## Abbreviations

CKD: Chronic kidney disease
eGFR: Glomerular filtration rate
IF: Impact factor
CVD: Cardiovascular disease
CV: Cardiovascular
SCRP: Serum C-reactive protein
IGF-1: Growth factor-1
MNT: Medical nutrition therapy
HD: Hemodialysis
PEW: Protein-energy wasting
GLIM: Global Leadership Initiative on Malnutrition
BMI: Body mass index
MIS: Malnutrition-inflammation score
SGA: Subjective global assessment (SGA)
